# On AI-augmented transformatizing in practice: protocols, quality criteria, and reporting standards for Human-AI Augmented Mixed Methods Research

**DOI:** 10.3389/fpsyg.2026.1933405

**Published:** 2026-10-02

**Authors:** Anthony J. Onwuegbuzie

**Affiliations:** Department of Educational Leadership and Management, University of Johannesburg, Johannesburg, South Africa

**Keywords:** AI-augmented transformatizing, Artificial intelligence, Human-AI Augmented Mixed Methods Research, Human-AI collaboration, methodological governance, Mixed Methods Research, recursive analysis, reporting standards

## Abstract

Artificial intelligence (AI) is increasingly reshaping qualitative research, quantitative research, and mixed methods research, but practical guidance for conducting, evaluating, and reporting human–AI analytic collaboration remains underdeveloped. Building on AI-augmented transformatizing, a representational–recursive framework that conceptualizes AI as a participant in analytic re-representation rather than merely as a technical tool, this article advances a practice-oriented framework for Human-AI Augmented Mixed Methods Research. The article offers three integrated contributions. First, it proposes the *AI-Augmented Transformatizing Protocol*, a recursive workflow for preparing representations, for assigning human and AI analytic roles, for generating AI-assisted transformations, for conducting human critique, for refining findings, and for constructing meta-inferences. Second, it introduces quality criteria for assessing methodological rigor in human-AI augmented analysis, including representational fidelity, prompt traceability, recursive transparency, human oversight integrity, algorithmic reflexivity, convergence-divergence interrogation, meta-inferential robustness, and ethical accountability. Third, it presents reporting standards designed to help authors disclose AI systems, model versions, prompting procedures, analytic iterations, researcher interventions, validation/legitimation strategies, ethical safeguards, and limitations. These proposed methodological components are conceptual and practice-oriented rather than empirically validated; accordingly, the worked example illustrates their operational logic, whereas future research is needed to establish their empirical performance, including the validity and reliability of the HAAM-QAM. Rather than treating AI use as an optional efficiency enhancement, the framework positions human-AI collaboration as a structured methodological practice requiring explicit design, documentation, reflexivity, and accountability. By translating the theory of AI-augmented transformatizing into protocols, quality criteria, and reporting standards, this article provides methodologists, researchers, authors, reviewers, editors, graduate educators, mentors, and the like with practical guidance for conducting transparent, rigorous, and ethically responsible AI-augmented mixed methods research in psychology and related fields. In so doing, it extends current reporting guidelines and quality frameworks by addressing the distinctive representational, recursive, and ethical demands introduced when AI participates directly in analysis.

## Bridging the theory–practice gap in Human-AI Augmented Mixed Methods Research

Artificial intelligence (AI) increasingly is becoming embedded within the design, analysis, interpretation, and dissemination of research across the social, behavioral, and health sciences. Machine learning and large language models (LLMs) now support pattern detection, classification, text generation, summarization, coding, and interpretation in ways that alter how researchers interact with empirical materials (Grimmer et al., 2022; Jordan and Mitchell, 2015). In both qualitative research and mixed methods research, these developments are especially consequential because AI systems do not merely accelerate existing analytic tasks; they also generate new representations that can shape meaning making and inference (Bender et al., 2021; Leonelli, 2019). Thus, the growing use of AI in research creates both epistemological challenges and methodological opportunities that require explicit guidance rather than ad hoc experimentation (Mittelstadt et al., 2016; Van Atteveldt et al., 2022).

Mixed methods research is particularly affected by the increasing participation of AI in analytic processes because its central methodological concern is integration. Integration involves relating, connecting, transforming, and interpreting qualitative research and quantitative research components/phases/strands in ways that produce insights greater than those yielded by either element alone (Fetters et al., 2013; Fetters and Freshwater, 2015). Yet, many established mixed methods research frameworks were developed before AI systems became active participants in analytic workflows, and, therefore, they do not fully address how algorithmically generated representations should be incorporated into integrative reasoning (Bryman, 2006; Greene et al., 1989). As a result, researchers need methodological procedures that specify how human and AI-generated representations can be compared, critiqued, transformed, and integrated responsibly (Grimmer et al., 2022; Van Atteveldt et al., 2022).

The article by Onwuegbuzie (2026), entitled, *AI-Augmented Transformatizing: A Representational–Recursive Framework for Human–AI Mixed Methods Analysis*, addressed this challenge at the conceptual level by introducing AI-augmented transformatizing as a representational–recursive framework for human–AI mixed methods analysis. AI-augmented transformatizing refers to the recursive, multidirectional transformation and re-transformation of qualitative, quantitative, and AI-generated representations through iterative human-AI interaction to generate increasingly integrated and robust meta-inferences. Within this framework, representations are understood as analytic forms that stand for phenomena rather than as the phenomena themselves, whereas meta-inferences represent higher order integrative conclusions that emerge from recursive interaction among multiple representations.

Onwuegbuzie’s (2026) manuscript conceptualized data as analytic representations, analysis as re-representation, integration as recursive interaction, and AI as a representational actor capable of participating in analytic transformation (Bogen and Woodward, 1988; Leonelli, 2019). It also advanced the claim that meta-inferences can emerge through recursive interaction between human-generated and AI-generated representations rather than through human interpretation alone (Fetters et al., 2013; Schoonenboom, 2022). However, because that article was primarily theoretical, it did not provide a comprehensive protocol, quality framework, or reporting checklist for researchers seeking to implement AI-augmented transformatizing in practice.

Therefore, the current follow-up article moves from theory to practice. Its central premise is that AI-augmented transformatizing cannot be treated simply as a helpful analytic technique, because any use of AI in mixed methods analysis affects representation, transformation, interpretation, reflexivity, and accountability (Bender et al., 2021; Mittelstadt et al., 2016). If AI-generated outputs are incorporated into qualitative coding, quantitative modeling, mixed methods integration, and/or meta-inference construction, then researchers must document how these outputs were produced, evaluated, revised, accepted, rejected, or transformed (O’Brien et al., 2014; Tong et al., 2007). Without such documentation, readers, reviewers, and editors cannot adequately assess the transparency, dependability, credibility, or legitimation of AI-augmented analyses (Lincoln and Guba, 1985; Onwuegbuzie and Hitchcock, 2019).

Existing reporting frameworks provide important foundations for enhancing research transparency, rigor, and reproducibility, but they are insufficient for the specific demands of Human-AI Augmented Mixed Methods Research. Over the last quarter of a century, and across the health, social, and behavioral sciences, numerous reporting standards have been developed to improve the completeness, transparency, and methodological quality of research reports. For example, the *Preferred Reporting Items for Systematic Reviews and Meta-Analyses* (*PRISMA*) Statement provides guidance for reporting systematic reviews and meta-analyses (Moher et al., 2009; Page et al., 2021); the *Sampling Strategy, Type of Study, Approaches, Range of Years, Limits, Inclusion and Exclusions, Terms Used, Electronic Sources* (*STARLITE*) Statement provides a structured framework for transparently reporting literature search strategies (Booth, 2006); the *Meta-analysis of Observational Studies in Epidemiology* (*MOOSE*) Statement offers reporting recommendations for meta-analyses of observational studies (Stroup et al., 2000); the *Strengthening the Reporting of Observational Studies in Epidemiology* (*STROBE*) Statement establishes standards for reporting observational studies (Von Elm et al., 2007); the *Consolidated Standards of Reporting Trials* (*CONSORT*) Statement promotes transparent reporting of randomized controlled trials (Schulz et al., 2010); the *Standards for Reporting Diagnostic Accuracy Studies* (*STARD*) Statement provides guidance for reporting diagnostic accuracy studies (Bossuyt et al., 2003); the *CAse REport* (*CARE*) Guidelines establish standards for reporting clinical case reports (Gagnier et al., 2013); the *Consolidated Health Economic Evaluation Reporting Standards* (*CHEERS*) Statement provides recommendations for reporting health economic evaluations (Husereau et al., 2013); and the *Statistical Analyses and Methods in the Published Literature* (*SAMPL*) Guidelines promote transparent reporting of statistical analyses (Lang and Altman, 2015).

Within qualitative inquiry, the *Consolidated Criteria for Reporting Qualitative Research* (*COREQ*) provide a comprehensive 32-item checklist for reporting interviews and focus groups (Tong et al., 2007), whereas the *Standards for Reporting Qualitative Research* (*SRQR*) offer broader guidance for transparent reporting across qualitative methodologies (O’Brien et al., 2014). Moreover, reporting guidance has been developed for qualitative evidence synthesis through the *Enhancing Transparency in Reporting the Synthesis of Qualitative Research* (*ENTREQ*) Statement (Tong et al., 2012) and for quality improvement studies through the *Standards for QUality Improvement Reporting Excellence* (*SQUIRE*) Guidelines (Davidoff et al., 2009). Collectively, these reporting frameworks demonstrate the scientific community’s longstanding recognition that methodological innovation should be accompanied by explicit standards that promote transparency, completeness, and reproducibility.

Beyond reporting guidelines, numerous appraisal tools have been developed to evaluate the conceptual, methodological, and reporting quality of research syntheses and empirical investigations. According to Hallinger (2014), quality appraisal encompasses three interrelated dimensions—conceptual quality, methodological quality, and reporting quality—that collectively determine the trustworthiness of research. Nevertheless, these dimensions frequently have been assessed in a fragmented manner, with most appraisal instruments emphasizing only one dimension of quality or focusing on a single methodological tradition (Onwuegbuzie and Sabates, 2025). Indeed, the *Enhancing the QUAlity and Transparency Of Health Research* (*EQUATOR*) *Network* currently provides more than 400 reporting guidelines and checklists spanning a wide range of research designs; however, the overwhelming majority of these guidelines were developed independently for specific study designs rather than for integrative methodological paradigms. Likewise, Rouleau et al. (2023) observed that methodological guidance remains uneven across systematic reviews of quantitative research, qualitative research, and mixed methods research, whereas Hong and Pluye (2019) argued that conceptual quality, methodological quality, and reporting quality are fundamentally interdependent and cannot be evaluated meaningfully in isolation. As Hong and Pluye (2019) further noted, deficiencies in reporting quality often impede valid assessments of both conceptual quality and methodological quality, underscoring the interconnected nature of these three dimensions. Similarly, Hallinger (2014) three-level analytical rubric represents one of the few multidimensional appraisal frameworks designed to evaluate systematic reviews simultaneously across conceptual, methodological, and reporting domains through eight quality criteria, specifically, the clarity of the study purpose, conceptual framework, search procedures, data extraction, data analysis, presentation of findings, discussion of limitations, and implications. Consequently, Hallinger (2014) framework demonstrates the value of integrating multiple dimensions of quality within a single evaluative system rather than treating them as independent constructs.

Building on multidimensional appraisal frameworks such as Hallinger (2014) analytical rubric, the field of mixed methods research likewise has generated important frameworks for evaluating methodological quality and integration. For example, the *Mixed Methods Appraisal Tool* (*MMAT*; Hong et al., 2018) provides criteria for appraising qualitative research, quantitative research, and mixed methods research studies within a unified evaluative framework. Similarly, integration frameworks developed by Fetters et al. (2013), Fetters and Freshwater (2015), together with both the original versions (Onwuegbuzie and Johnson, 2006) and expanded versions (Onwuegbuzie et al., 2026) of the *mixed methods legitimation framework*, provide guidance for evaluating the quality, coherence, rigor, and integration of qualitative research and quantitative research components, phases, or strands.

Notably, the late Emeritus Professor M. Teresa Anguera and her colleagues provide another important integrative tradition within mixed methods research, including explicit attention to best-practice approaches in psychological science (Anguera et al., 2020). Particularly through systematic observational methodology, this work demonstrates how qualitative information can be transformed into quantitatively analyzable representations and subsequently returned to qualitative interpretation. Their QUAL→QUAN→QUAL logic illustrates how qualitative and quantitative elements can be connected through successive transformations rather than treated as separate analytic strands, while emphasizing methodological rigor, data quality, and explicit integration (Anguera et al., 2017, 2023). Recent applications further illustrate how this approach can identify patterns within systematically coded qualitative material and integrate those quantitative results into contextually grounded interpretation (Tronchoni et al., 2024). This work is especially relevant to the present framework because it demonstrates an established methodological precedent for treating transformation between representational forms as a mechanism of mixed methods integration.

More recently, Hallinger (2014) eight-criterion analytical rubric for evaluating systematic reviews has been reconceptualized and extended by Onwuegbuzie and Sabates (2025) into an integrated multidimensional quality appraisal framework applicable across systematic quantitative reviews, systematic qualitative reviews, systematic mixed methods reviews, and reviews integrating multiple research traditions. Building on Hallinger (2014) original rubric, Onwuegbuzie and Sabates (2025) explicitly organized eight quality criteria into three interdependent dimensions—conceptual quality, methodological quality, and reporting quality—and operationalized these dimensions through a *Systematic Review Quality Matrix* designed to provide a unified evaluation of systematic reviews across methodological traditions. This reconceptualization represents an important step toward integrated quality appraisal because it recognizes that conceptual, methodological, and reporting quality should be evaluated simultaneously rather than independently.

More recent research has begun to examine generative AI directly within both qualitative research and mixed methods research. Empirical comparisons of human and AI-assisted qualitative analysis indicate that generative AI can accelerate coding and theme generation and sometimes identify useful alternative patterns, but also can produce inconsistent, decontextualized, or insufficiently nuanced interpretations, reinforcing the need for human verification and interpretive judgment (Friedman et al., 2025; Kabir et al., 2025). Related work has shown that the methodological value of AI-assisted qualitative analysis depends substantially on prompt design, iterative interaction, and systematic validation of AI-generated interpretations rather than on uncritical acceptance of model outputs (Combrinck, 2024; Kabir et al., 2025). Within mixed methods research, emerging applications likewise demonstrate both the possibilities and methodological responsibilities associated with generative AI: Combrinck (2024) found that AI-assisted qualitative coding improved when the model was provided with human-generated examples and that researcher judgment remained necessary for quantitative analysis and integrative analysis, whereas Ramlo (2025) demonstrated how ChatGPT can participate in integrated data collection within Q methodology. Consistent with these developments, James et al. (2024) called explicitly for transparent disclosure of AI and automated-tool use in mixed methods studies. Taken together, this emerging literature shifts the methodological question from whether generative AI can perform research tasks to how its contributions should be structured, evaluated, documented, and integrated while preserving human interpretive responsibility (Combrinck, 2024; Friedman et al., 2025; James et al., 2024; Kabir et al., 2025; Ramlo, 2025).

Collectively, these frameworks substantially have advanced standards for designing, conducting, appraising, and reporting mixed methods research. Nevertheless, they were developed before LLMs and other forms of generative AI became active participants in analytic workflows. Consequently, they provide little or no guidance regarding AI-specific methodological issues such as prompt engineering, documentation of prompts, disclosure of model architecture and versioning, documentation of iterative Human-AI interactions, evaluation of AI-generated representations, management of algorithmic bias, auditing of human oversight, or standards for reporting AI-assisted integrated conclusions (Bender et al., 2021; Mehrabi et al., 2021). Thus, although existing reporting standards, quality appraisal tools, and methodological frameworks provide an indispensable foundation for rigorous research, none currently offers a comprehensive methodological framework for documenting, evaluating, and reporting the recursive human-AI analytic processes that characterize AI-augmented transformatizing. Accordingly, there is a pressing need for an integrated methodological framework that specifies not only how AI-augmented mixed methods research should be conducted, but also how it should be evaluated and reported. The present article addresses this need by proposing protocols, quality criteria, and reporting standards for AI-augmented transformatizing in Human-AI Augmented Mixed Methods Research.

A practice-oriented framework is needed because AI-augmented analysis introduces new forms of methodological vulnerability. AI outputs can appear fluent, coherent, and authoritative even when they reflect training-data bias, model opacity, hallucination, or decontextualized pattern recognition (Bender et al., 2021; Mehrabi et al., 2021). Human researchers also can over-rely on AI-generated representations, especially when outputs are produced quickly, formatted persuasively, or aligned with researchers’ expectations (Mittelstadt et al., 2016; Noble, 2018). Consequently, rigorous AI-augmented mixed methods research requires procedures that preserve human interpretive responsibility while making AI participation visible, auditable, and contestable (Barocas et al., 2023; Lincoln and Guba, 1985).

This article introduces three interrelated contributions designed to operationalize AI-augmented transformatizing. First, it proposes the *AI-Augmented Transformatizing Protocol*, a recursive workflow for preparing representations, for assigning human and AI analytic roles, for generating AI-assisted transformations, for conducting human critique, for refining findings, and for constructing meta-inferences (Fetters et al., 2013; Schoonenboom, 2022). Second, it advances quality criteria for evaluating the integrity, transparency, human oversight, and methodological rigor of Human-AI augmented mixed methods analysis (Lincoln and Guba, 1985; Onwuegbuzie and Hitchcock, 2019). Third, it presents reporting standards that specify what researchers should disclose when AI participates in data transformation, data analysis, data interpretation, integration, and/or reporting (O’Brien et al., 2014; Tong et al., 2007).

This article is written for methodologists, researchers, authors, reviewers, editors, graduate educators, mentors, and the like who need practical guidance for responsible AI use in mixed methods research. I do not argue that AI should replace human interpretation, nor do I treat AI-generated outputs as being epistemically superior to human analysis (Bender et al., 2021; Leonelli, 2019). Rather, I position human-AI collaboration as a structured, reflexive, and accountable methodological practice in which human researchers retain responsibility for framing, interpretation, ethical judgment, and final meta-inference (Lincoln and Guba, 1985; Mittelstadt et al., 2016). In so doing, the article extends AI-augmented transformatizing from a theoretical framework into a usable methodological system for psychological science and beyond.

## From conceptual framework to methodological practice

The companion article, *AI-Augmented Transformatizing: A Representational–Recursive Framework for Human–AI Mixed Methods Analysis* (Onwuegbuzie, 2026), provides the conceptual point of departure for the present methodological framework. However, in order to ensure that the present article can be understood independently, its essential constructs are summarized here. *AI-augmented transformatizing* refers to the recursive, multidirectional transformation and re-transformation of qualitative, quantitative, integrated, and AI-generated representations through iterative Human-AI interaction to support increasingly integrated and defensible meta-inferences. A *representation* is an analytic form—such as a qualitative theme, quantitative result, joint display, narrative interpretation, or AI-generated synthesis—that stands for some aspect of the phenomenon under investigation rather than reproducing that phenomenon directly (Bogen and Woodward, 1988; Leonelli, 2019). *Re-representation* occurs when an existing representation is transformed into a new analytic form or interpretation, whether by a human researcher, an AI system, or their recursive interaction. *Recursion* denotes the iterative process through which these representations are generated, compared, critiqued, revised, and re-entered into subsequent analytic cycles rather than being integrated through a single terminal step. Within this process, AI functions as an *analytic participant* by generating provisional representations that can expand the available interpretive space but that remain subject to human evaluation rather than being treated as authoritative conclusions (Bender et al., 2021; Mittelstadt et al., 2016). Finally, *meta-inference* refers to the higher-order integrative interpretation constructed by human researchers through the systematic examination of convergence, complementarity, and divergence across recursively developed representations (Fetters et al., 2013; Schoonenboom, 2022). Human researchers retain interpretive authority throughout this process: they frame the inquiry, determine the methodological role assigned to AI, evaluate competing representations, make ethical and theoretical judgments, and assume responsibility for final meta-inferences. These constructs constitute the conceptual vocabulary required for the protocol, quality criteria, and reporting standards developed below; accordingly, understanding or evaluating the present methodological framework does not require access to the companion article.

Although the theoretical framework clarifies *why* AI can participate in mixed methods analysis, it does not specify *how* researchers should organize, document, evaluate, or report recursive human-AI collaboration in empirical research studies. Existing literature offers valuable guidance on mixed methods integration and AI-assisted inquiry but provides comparatively little practical direction regarding recursive interaction, prompt documentation, evaluation of AI-generated representations, preservation of reflexivity, maintenance of human accountability, and transparent reporting of AI participation (Bender et al., 2021; Grimmer et al., 2022; Van Atteveldt et al., 2022).

The purpose of the present article is to address this methodological gap by translating AI-augmented transformatizing into an operational methodology. Specifically, this article introduces an *AI-Augmented Transformatizing Protocol* for structuring recursive human-AI analysis, proposes quality criteria for evaluating the rigor and trustworthiness of AI-augmented mixed methods research, and presents reporting standards that make human-AI analytic processes transparent, reproducible, and open to critical appraisal. In so doing, it extends AI-augmented transformatizing from a conceptual framework to a practical methodological system for conducting rigorous Human-AI Augmented Mixed Methods Research.

## Conceptualizing Human-AI Augmented Mixed Methods Research

For operational purposes, these conceptual foundations are organized around five interrelated methodological principles: (a) AI as an analytic participant, (b) complementary representational roles for human researchers and AI systems, (c) distributed analytic agency, (d) human interpretive authority, and (e) recursive Human-AI collaboration.

Together, these principles distinguish the respective roles and responsibilities of human researchers and AI systems. AI contributes provisional analytic representations and alternative interpretive possibilities, whereas human researchers contribute contextual, theoretical, methodological, and ethical judgment. Therefore, analytic activity can be distributed across Human-AI interaction, but interpretive authority and responsibility for final methodological decisions and meta-inferences remain with human researchers. Collaboration is recursive when evaluation of one representation informs subsequent prompting, critique, refinement, and integration. Collectively, these principles provide the conceptual foundation for operationalizing the protocol, quality criteria, and reporting standards that follow.

Figure 1 summarizes this conceptual architecture. The upper portion depicts the five foundational principles that characterize Human-AI Augmented Mixed Methods Research, whereas the lower portion illustrates how these principles are operationalized through the AI-Augmented Transformatizing Protocol, evaluated using the Human-AI Augmented Mixed Methods Quality Assessment Matrix, and documented using the reporting standards introduced in subsequent sections. Thus, the figure serves as the conceptual bridge between the theoretical framework and the methodological guidance that follows.

**FIGURE 1:**
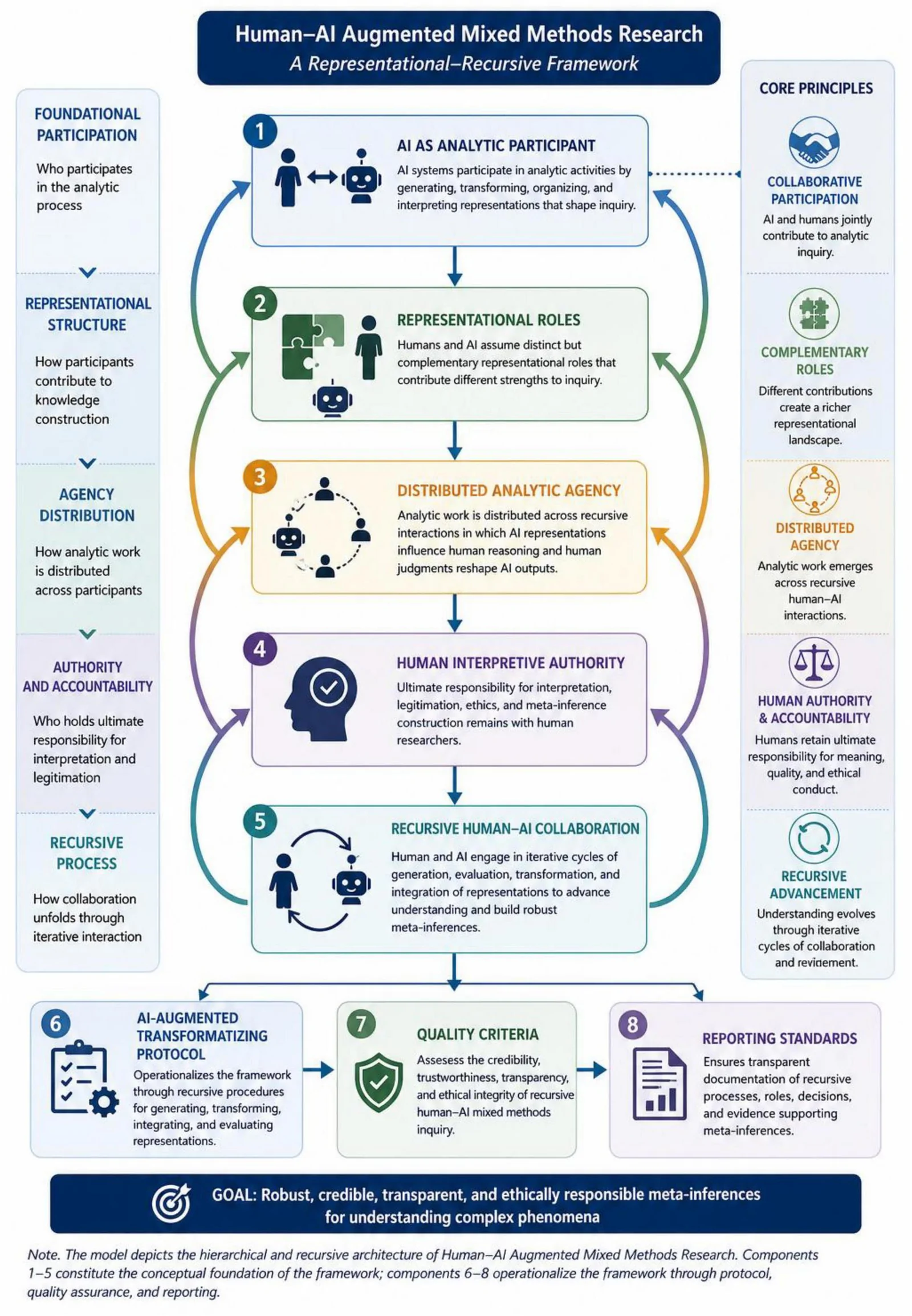
Conceptual architecture of Human-AI Augmented Mixed Methods Research. Solid downward arrows represent the hierarchical progression from conceptual foundations to methodological implementation. Curved bidirectional arrows represent the recursive interactions that characterize Human-AI collaboration throughout the analytic process. The Figure should be interpreted as depicting a recursive rather than strictly linear process, with successive cycles of interaction occurring throughout the conduct of Human-AI Augmented Mixed Methods Research. The model depicts the hierarchical and recursive architecture of Human-AI augmented Mixed Methods Research. Components 1–5 constitute the conceptual foundation of the framework; components 6–8 operationalize the framework through protocol, quality assurance, and reporting.

Accordingly, the remainder of this article focuses on operational implementation. Specifically, the article presents the AI-Augmented Transformatizing Protocol, introduces quality criteria for evaluating Human-AI Augmented Mixed Methods Research, and proposes reporting standards designed to ensure transparency, reproducibility, and methodological accountability.

## The AI-Augmented Transformatizing Protocol

### Overview of the AI-Augmented Transformatizing Protocol

The companion article established the conceptual foundations of AI-Augmented Transformatizing by proposing a representational-recursive framework for Human-AI collaboration in mixed methods research (Onwuegbuzie, 2026). The present article translates that conceptual framework into an operational methodology by specifying how researchers can conduct, document, evaluate, and report recursive Human-AI collaboration during empirical inquiry.

The AI-Augmented Transformatizing Protocol consists of seven recursive phases that guide investigators from the preparation of empirical representations through the construction and validation of meta-inferences. These phases are (a) Phase 1: Representational Preparation; (b) Phase 2: Human Analytic Orientation; (c) Phase 3: AI-Assisted Transformation; (d) Phase 4: Human Critique and Contextualization; (e) Phase 5: Recursive Re-Prompting and Re-Representation; (f) Phase 6: Meta-Inference Construction; and (g) Phase 7: Reflexive Audit and Validation. Although presented sequentially for clarity, the protocol is *inherently iterative*. Researchers may revisit earlier phases whenever emerging representations, competing interpretations, or methodological concerns require further refinement. Thus, the protocol should be understood as a recursive analytic framework rather than a fixed sequence of procedural steps.

Each phase has a distinct methodological purpose while contributing to the overall objective of producing transparent, rigorous, and ethically accountable Human-AI Augmented Mixed Methods Research. Together, the seven phases establish procedures for preparing representations, structuring Human-AI collaboration, evaluating AI-generated representations, refining interpretations through iterative interaction, constructing defensible integrated conclusions, and documenting the complete analytic process.

Table 1 summarizes the seven phases of the AI-Augmented Transformatizing Protocol, including the primary purpose, principal researcher activities, AI contributions, and expected outputs associated with each phase. The remainder of this section briefly describes each phase of the protocol.

**TABLE 1:** The Artificial intelligence (AI)-Augmented Transformatizing Protocol for human-AI augmented mixed methods research.

Phase	Purpose	Human role	AI role	Primary output
1	Prepare representations	Organize corpus	None	Prepared corpus
2	Establish analytic orientation	Define theory and prompts	None	Analytic charter
3	Generate alternative representations	Prompt and supervise	Generate representations	AI representations
4	Critique representations	Evaluate	Respond to critique	Contextualized representations
5	Recursive refinement	Revise prompts	Generate revisions	Refined representations
6	Construct meta-inferences	Integrate	Support synthesis	Meta-inferences
7	Audit and validate	Evaluate process	Provide documentation	Audit report

The protocol comprises seven recursive phases that guide researchers from representational preparation through reflexive audit and validation. Although presented sequentially, the phases are inherently iterative and may be revisited as representations evolve during Human-AI collaboration.

### Phase 1: representational preparation

**Purpose:** The purpose of Phase 1 is to prepare empirical representations for recursive Human-AI analysis. Rather than viewing qualitative data and quantitative data simply as information to be analyzed, the protocol treats them as representations that subsequently will undergo transformation, comparison, and integration.

**Core activities:** Researchers identify all representations relevant to the investigation, including qualitative materials, quantitative datasets, integrated displays, documentary evidence, and contextual information. They document the provenance of each representation, verify data integrity, remove identifying information where necessary, and ensure that contextual information required for subsequent interpretation is preserved. Researchers also prepare an initial analytic memorandum documenting the research questions, theoretical orientation, and preliminary assumptions that will inform later stages of the protocol.

**Output:** The outcome of Phase 1 is a representationally prepared analytic corpus with documented provenance, contextual information, and initial analytic orientation.

### Phase 2: human analytic orientation

**Purpose:** Phase 2 establishes the conceptual and methodological framework that will guide recursive Human-AI collaboration.

**Core activities:** Researchers explicitly articulate the purpose of the investigation, theoretical perspective, methodological approach, mixed methods research design, and intended role of AI throughout the analysis. They develop an AI interaction plan specifying anticipated prompting strategies, documentation procedures, and quality safeguards while identifying potential sources of human and algorithmic bias. These decisions are recorded within a Recursive Analytic Charter that serves as the guiding document throughout the investigation.

**Output:** The outcome is an explicitly documented analytic framework that establishes the conceptual and methodological foundations for subsequent Human-AI interaction.

### Phase 3: AI-assisted transformation

**Purpose:** Phase 3 introduces AI as an analytic participant by generating alternative representations that expand the interpretive possibilities available for subsequent human evaluation.

**Core activities:** Researchers develop and document prompts that specify the analytic objectives, contextual information, and desired representational outputs. AI systems generate coding structures, thematic organizations, conceptual summaries, statistical interpretations, explanatory narratives, or other representations appropriate to the investigation. Whenever appropriate, researchers request multiple alternative representations rather than a single response to encourage analytic diversity. Every prompt, response, model version, configuration, and interaction is documented to ensure that prompts and resulting representations can be traced to their documented sources and interaction histories.

**Output:** The outcome is a documented corpus of AI-generated representations that serves as the foundation for recursive human evaluation.

### Phase 4: human critique and contextualization

**Purpose:** Phase 4 operationalizes human interpretive authority by evaluating AI-generated representations before they contribute to subsequent analysis.

**Core activities:** Researchers assess each AI-generated representation for representational fidelity, contextual appropriateness, theoretical coherence, methodological consistency, and ethical acceptability. Representations may be accepted, modified, integrated with alternative representations, or rejected. Throughout this process, researchers maintain critique memoranda documenting the rationale underlying each evaluative decision.

**Output:** The outcome is a corpus of critically evaluated representations whose strengths, limitations, and methodological status have been documented explicitly.

### Phase 5: recursive re-prompting and re-representation

**Purpose:** Phase 5 refines analytic understanding through successive cycles of Human-AI interaction.

**Core activities:** Researchers revise prompts in response to insights generated during Phase 4 and generate refined representations that address conceptual ambiguities, alternative interpretations, or methodological concerns. Recursive cycles continue until additional iterations no longer produce substantively meaningful analytic refinement. Throughout this process, researchers preserve a transparent record of how representations develop across successive iterations.

**Output:** The outcome is a refined set of representations supported by a complete record of how prompts and representations developed across iterations.

### Phase 6: meta-inference construction

**Purpose:** Phase 6 integrates recursively refined representations into coherent meta-inferences.

**Core activities:** Researchers compare qualitative, quantitative, integrated, AI-generated, and recursively refined representations to identify convergent findings, divergent interpretations, theoretical implications, and explanatory relationships. Proposed meta-inferences are evaluated for methodological defensibility, empirical support, theoretical coherence, and transparency before being accepted. Human researchers retain responsibility for all final interpretive judgments.

**Output:** The outcome is a rigorously justified set of meta-inferences supported by explicit representational evidence and methodological documentation.

### Phase 7: reflexive audit and validation

**Purpose:** The final phase evaluates the integrity and transparency of the complete recursive Human-AI analytic process.

**Core activities:** Researchers conduct a comprehensive methodological audit examining prompt histories, AI interactions, the sources and development of representations, critique memoranda, iterative modifications, reflexive documentation, and justification of the final integrated conclusions. They evaluate whether human interpretive authority has been maintained, methodological decisions documented transparently, and AI participation governed consistently throughout the investigation. Study limitations, methodological adaptations, and lessons learned also are documented.

**Output:** The outcome is a Recursive Methodological Audit Report that provides a transparent audit trail supporting the credibility, reproducibility, and trustworthiness of the investigation.

The following section builds on this operational framework by introducing the Human-AI Augmented Mixed Methods Quality Assessment Matrix (HAAM-QAM), which specifies the criteria by which the rigor and trustworthiness of AI-Augmented Transformatizing should be evaluated.

## Quality criteria for human-AI augmented mixed methods analysis

### Why new quality criteria are needed

The AI-Augmented Transformatizing Protocol presented in the preceding section specifies how Human-AI Augmented Mixed Methods Research should be conducted through recursive collaboration between human researchers and AI systems. However, methodological procedures alone are insufficient for ensuring scientific rigor. Every mature methodological tradition requires explicit criteria by which research quality can be evaluated, compared, and improved (Hong and Pluye, 2019; Onwuegbuzie and Sabates, 2025). As AI assumes an increasingly active role in generating analytic representations, existing frameworks for evaluating qualitative research, quantitative research, and mixed methods research require extension to address the distinctive methodological challenges associated with recursive Human-AI collaboration.

Traditional quality frameworks emphasize issues such as validity, trustworthiness, credibility, transparency, and integration (Lincoln and Guba, 1985; O’Cathain, 2010; Onwuegbuzie and Johnson, 2006). Although these criteria remain essential, they were developed under the assumption that analytic reasoning resides (almost) exclusively with human investigators. Human-AI Augmented Mixed Methods Research introduces additional methodological considerations, including documentation of prompts, the sources and development of representations, iterative Human-AI interaction, algorithmic influences, and the preservation of human interpretive authority (Bender et al., 2021; Mittelstadt et al., 2016). Consequently, evaluating AI-augmented inquiry requires attention not only to research outcomes, but also to the recursive processes through which human and AI-generated representations are developed, critiqued, refined, and integrated.

Accordingly, this section introduces the Human-AI Augmented Mixed Methods Quality Assessment Matrix (HAAM-QAM), a multidimensional framework for evaluating the methodological rigor of AI-augmented mixed methods inquiry. Rather than replacing established standards for qualitative research, quantitative research, or mixed methods research, the HAAM-QAM extends those traditions by incorporating quality dimensions specifically required when AI participates in recursive knowledge construction.

The HAAM-QAM also intersects with broader international frameworks for responsible AI governance. UNESCO’s Recommendation on the Ethics of Artificial Intelligence emphasizes transparency and explainability, responsibility and accountability, human oversight, privacy and data protection, fairness, and mechanisms for audit and traceability (UNESCO, 2022). Similarly, the Organization for Economic Co-operation and Development (OECD) AI Principles emphasize human agency and oversight, transparency and responsible disclosure, robustness and safety, accountability, and traceability across the AI system lifecycle (Organisation for Economic Co-operation and Development, 2024), whereas the European Union Artificial Intelligence Act establishes risk-based governance requirements that include transparency, documentation, accountability, and human oversight for relevant AI systems (European Parliament and Council of the European Union, 2024). These governance principles converge with several HAAM-QAM dimensions, particularly Prompt Traceability, Recursive Transparency, Human Oversight Integrity, Algorithmic Reflexivity, and Ethical Accountability. However, the HAAM-QAM applies these broader governance commitments specifically to methodological quality in Human-AI Augmented Mixed Methods Research by translating them into criteria for evaluating how AI-generated representations are produced, scrutinized, integrated, and reported during empirical inquiry. Thus, the matrix should be understood not as replacing established AI-governance principles, but as operationalizing related commitments within the particular methodological context of AI-assisted research.

### Foundational principles of quality evaluation

The HAAM-QAM translates the preceding methodological principles into five evaluative commitments: representational integrity, recursive transparency, human interpretive authority, methodological transparency, and epistemological accountability. Together, these commitments require researchers to preserve the provenance and integrity of representations, to document how analyses develop across iterative Human-AI interactions, to retain human responsibility for methodological and interpretive judgments, to make AI-assisted procedures open to scrutiny, and to justify resulting knowledge claims through transparent evidentiary pathways (Bogen and Woodward, 1988; Hong and Pluye, 2019; Leonelli, 2019; Mittelstadt et al., 2016). Rather than restating the conceptual principles developed earlier, these commitments provide the immediate evaluative basis for the HAAM-QAM domains and dimensions presented below.

### The human-AI augmented mixed methods quality assessment matrix

Building on these five principles, the Human-AI Augmented Mixed Methods Quality Assessment Matrix (HAAM-QAM) organizes methodological quality into three complementary domains—Representational Quality, Analytic Quality, and Meta-Inferential Quality—encompassing eight interrelated quality dimensions. Rather than functioning as a numerical scoring instrument, the matrix is intended to guide systematic methodological reflection, peer review, editorial evaluation, and researcher self-assessment throughout the research process. At this stage, however, the HAAM-QAM should be regarded as a proposed methodological appraisal framework rather than as an empirically validated measurement instrument. Its domains, dimensions, and performance descriptors require systematic empirical evaluation, including studies of construct-related validity and inter-rater reliability, before the matrix can be interpreted as having established measurement properties. Table 2 organizes the three quality domains together with their corresponding quality dimensions into an integrated framework for evaluating the methodological rigor, transparency, and trustworthiness of Human-AI Augmented Mixed Methods Research.

**TABLE 2:** The Human-AI Augmented Mixed Methods Quality Assessment Matrix (HAAM-QAM).

Domain	Quality dimension	Primary evaluation question	Emerging	Developing	Proficient	Exemplary
Representational quality	Representational fidelity	To what extent do representations preserve empirical meaning throughout recursive transformation?	Numerous distortions or undocumented transformations	Fidelity partially demonstrated	Representations consistently preserve empirical meaning	Representational integrity documented comprehensively across all recursive cycles
	Prompt traceability	To what extent can every AI-generated representation be traced to documented prompts?	Prompt documentation absent or incomplete	Partial documentation	Complete prompt history maintained	Fully version-controlled recursive prompt history with complete provenance
	Recursive transparency	To what extent is the recursive analytic process completely auditable?	Major recursive decisions undocumented	Partial audit trail	Comprehensive audit trail maintained	Complete recursive lineage documented across all protocol phases
Analytic quality	Human oversight integrity	To what extent did humans retain responsibility for all substantive analytic decisions?	AI assumed substantial analytic authority	Human oversight inconsistent	Human oversight consistently documented	Human interpretive authority explicitly demonstrated throughout all recursive cycles
	Algorithmic reflexivity	To what extent were algorithmic influences recognized and evaluated critically?	Algorithmic influences ignored	Limited discussion of AI limitations	Algorithmic influences examined systematically	Continuous recursive reflexivity regarding both human and algorithmic influences
	Convergence–divergence interrogation	To what extent were convergent and divergent representations investigated rigorously?	Divergence largely ignored	Limited exploration of disagreement	Systematic examination of convergence and divergence	Recursive interrogation generated novel theoretical insights from both convergence and divergence
Meta-inferential quality	Meta-inferential robustness	To what extent are the final meta-inferences recursively justified and well-supported?	Weak evidentiary support	Moderate evidentiary support	Strong recursively integrated evidentiary support	Comprehensive Meta-Inference Portfolio demonstrating recursive justification
	Ethical accountability	To what extent was Human-AI collaboration conducted and reported ethically?	Ethical issues minimally addressed	Basic ethical safeguards documented	Ethical governance documented comprehensively	Continuous ethical stewardship integrated throughout every recursive phase

#### Domain I: representational quality

Representational quality evaluates the integrity of the representational system that underlies recursive human-AI inquiry. It comprises three dimensions, as follows:

- Representational fidelity evaluates whether qualitative, quantitative, integrated, and AI-generated representations remain faithful to the empirical phenomena, theoretical orientation, and contextual circumstances from which they were derived.
- Prompt traceability assesses the extent to which AI-generated representations can be linked explicitly to documented prompts, model configurations, contextual inputs, and recursive interaction histories.
- Recursive transparency evaluates whether the complete history of representational development—including prompt evolution, critique memoranda, methodological adaptations, and recursive refinements—has been documented sufficiently to permit methodological reconstruction and critical appraisal.

Together, these dimensions ensure that the representational foundations of Human-AI inquiry remain transparent, traceable, and methodologically defensible.

#### Domain II: analytic quality

Analytic Quality evaluates the integrity of recursive human-AI collaboration during analysis. It focuses on the processes through which representations are generated, evaluated, refined, and integrated. It comprises three dimensions, as follows:

- **Human oversight integrity** assesses whether researchers maintained continuous responsibility for methodological decisions, representational adjudication, interpretation, and meta-inference construction.
- **Algorithmic reflexivity** evaluates the extent to which researchers critically examined the influence of AI systems, prompt design, model limitations, and potential algorithmic bias throughout recursive inquiry.
- **Convergence-divergence interrogation** examines whether convergent and divergent representations were investigated systematically before meta-inferences were constructed. Divergence is viewed not as methodological failure, but as an opportunity for deeper theoretical understanding and recursive refinement.

Collectively, these dimensions evaluate whether Human-AI collaboration has been conducted rigorously while preserving human interpretive authority and methodological accountability.

#### Domain III: meta-inferential Quality

The final domain evaluates the quality of the knowledge claims produced through recursive human-AI collaboration. It comprises two dimensions, as follows:

- Meta-inferential robustness assesses whether final meta-inferences are supported by multiple complementary representations, theoretically coherent, empirically justified, and transparently linked to the evidentiary pathway through which they were developed.
- Ethical accountability evaluates whether AI participation has been disclosed transparently, potential algorithmic biases have been addressed appropriately, confidentiality has been protected, and researchers have retained responsibility for all published interpretations and conclusions.

Together, these dimensions ensure that Human-AI Augmented Mixed Methods Research produces knowledge claims that are not only methodologically rigorous, but also ethically responsible and scientifically trustworthy.

### Interpreting the HAAM-QAM

The Human-AI Augmented Mixed Methods Quality Assessment Matrix (Table 2) should be interpreted holistically rather than mechanically. Investigators should not assume that strengths within one quality dimension compensate for weaknesses in another because each dimension addresses a distinct aspect of recursive Human-AI inquiry. Rather than functioning as independent evaluative criteria, the eight quality dimensions operate as an interconnected methodological system in which representational quality, analytic quality, and meta-inferential quality mutually influence one another.

Likewise, the four performance levels presented in Table 2—Emerging, Developing, Proficient, and Exemplary—are intended to stimulate methodological reflection rather than to generate numerical quality scores. Assigning numerical weights to the dimensions would imply that methodological rigor is additive rather than recursive, a position inconsistent with the representational-recursive framework advanced throughout this article. Instead, researchers, reviewers, and editors should evaluate the overall pattern of strengths and limitations across the three quality domains when assessing the methodological integrity of Human-AI Augmented Mixed Methods Research.

The HAAM-QAM is intended for use throughout the research process. During study design, it can identify potential methodological vulnerabilities; during analysis, it can guide recursive monitoring of Human-AI interaction; and following study completion, it can support peer review, editorial evaluation, graduate supervision, and methodological self-assessment. Accordingly, the framework functions simultaneously as a planning tool, a monitoring framework, and a comprehensive quality appraisal system.

The next section builds directly on the HAAM-QAM by introducing reporting standards designed to ensure that Human-AI Augmented Mixed Methods Research is communicated with sufficient transparency, traceability, and accountability to enable critical evaluation, methodological replication, and responsible scientific use.

## Reporting standards for AI-augmented transformatizing in Human-AI Augmented Mixed Methods Research

### Why Human-AI Augmented Mixed Methods Research requires new reporting standards

The preceding sections introduced the AI-Augmented Transformatizing Protocol and the Human-AI Augmented Mixed Methods Quality Assessment Matrix (HAAM-QAM), thereby providing methodological guidance for conducting and evaluating Human-AI Augmented Mixed Methods Research, respectively. However, rigorous methodology alone is insufficient if the recursive processes through which Human-AI collaboration occurs are not reported transparently. Readers, reviewers, and editors can evaluate methodological quality only when investigators provide sufficient documentation to understand how AI participated in the research process and how human researchers maintained interpretive authority throughout recursive inquiry (Hong and Pluye, 2019; O’Brien et al., 2014; Onwuegbuzie and Sabates, 2025).

Existing reporting guidelines—such as PRISMA, CONSORT, COREQ, and SRQR—substantially have improved the transparency of qualitative research, quantitative research, and mixed methods research by promoting comprehensive reporting of study design, analytic procedures, and research findings (Page et al., 2021; Tong et al., 2007). Nevertheless, as noted previously, these frameworks were developed before generative AI became an active participant in scientific inquiry and, therefore, provide limited guidance regarding AI system disclosure, prompt documentation, recursive Human-AI interaction, algorithmic influences, or AI-specific methodological accountability (Bender et al., 2021; Van Atteveldt et al., 2022).

Accordingly, Human-AI Augmented Mixed Methods Research requires complementary reporting standards that make recursive Human-AI collaboration visible, transparent, and open to critical evaluation. These standards are intended to complement rather than to replace existing reporting guidelines by documenting those aspects of AI-assisted inquiry that are unique to recursive Human-AI collaboration.

### Principles underlying reporting transparency

The reporting framework proposed in this article is grounded in six interrelated principles that collectively support transparent and trustworthy Human-AI scholarship. These principles are transparency, reproducibility, traceability, reflexivity, accountability, and auditability. Together, they establish the conceptual foundation for reporting Human-AI Augmented Mixed Methods Research in ways that make recursive analytic processes visible, critically appraisable, and methodologically accountable. The following descriptions illustrate how each principle contributes to transparent and trustworthy Human-AI Augmented Mixed Methods Research:

- Transparency requires explicit documentation of AI participation throughout the research process.
- Reproducibility emphasizes comprehensive methodological reporting sufficient to permit informed reconstruction and critical evaluation of recursive inquiry, recognizing that identical AI outputs may not always be reproducible because of evolving AI systems.
- Traceability requires that AI-generated representations remain linked to the prompts, model configurations, and recursive interactions responsible for their generation.
- Reflexivity extends conventional researcher reflexivity by encouraging investigators to consider both human and algorithmic influences on knowledge construction.
- Accountability requires that responsibility for methodological decisions, interpretation, and published conclusions remain explicitly with human researchers.
- Auditability requires preserving sufficient documentation to permit independent evaluation of the recursive analytic process.

Together, these principles establish reporting as a methodological record of Human-AI collaboration rather than merely a retrospective description of completed research.

### Reporting standards

The proposed reporting standards are organized into six complementary reporting domains corresponding to the principal components of AI-Augmented Transformatizing. These domains are AI System Disclosure, Prompting Procedures, Human-AI Interaction, Validation and Audit Trail, Ethical Safeguards, and Limitations of AI Use. Together, these reporting domains provide a comprehensive framework for documenting Human-AI collaboration in ways that promote transparency, reproducibility, accountability, and methodological scrutiny.

#### AI system disclosure

Researchers should report the AI platform, developer, model family, model version, access date, system configuration, and analytic role assigned to AI throughout the investigation. These disclosures establish the computational context within which human-AI collaboration occurred and enable readers to evaluate the technological environment in which analytic representations were generated. Therefore, comprehensive AI system disclosure strengthens methodological transparency by ensuring that AI-assisted analyses can be interpreted and critically appraised within their appropriate computational context.

#### Prompting procedures

Transparent reporting requires documentation of prompt development, prompt evolution, recursive prompting, and contextual information supplied to AI systems. Because prompts function as methodological interventions rather than simple technical instructions, readers should be able to understand how prompting influenced the development of interpretations across iterations. Therefore, comprehensive documentation of prompting procedures enables readers to evaluate how Human-AI interactions shaped analytic development and the resulting integrated conclusions.

#### Human-AI interaction

Researchers should describe the recursive interaction cycles characterizing human-AI collaboration, including researcher interventions, acceptance, rejection, or modification of AI-generated representations, and the methodological rationale underlying major recursive decisions. Such reporting demonstrates that AI functioned as an analytic participant operating under continuous human supervision rather than as an autonomous analytic agent. Therefore, comprehensive documentation of Human-AI interactions enables readers to evaluate how iterative collaboration contributed to analytic refinement, methodological decision making, and the development of defensible integrated conclusions.

#### Validation and audit trail

Investigators should document validation/legitimation procedures, version histories, recursive audit trails, methodological records, and other materials necessary to evaluate the integrity of the recursive analytic process. Because human-AI collaboration evolves across successive analytic cycles, documentation should extend beyond final findings to include the methodological pathway through which analyses and integrated conclusions developed. Therefore, comprehensive validation and audit documentation enables readers, reviewers, and editors to evaluate the credibility, dependability, and methodological integrity of Human-AI Augmented Mixed Methods Research.

#### Ethical Safeguards

Researchers should report procedures used to protect participant confidentiality, to evaluate and to mitigate algorithmic bias, to verify AI-generated representations, to ensure responsible AI use, and to preserve human interpretive authority. Ethical reporting should demonstrate that AI participation occurred within clearly defined methodological and ethical boundaries. Therefore, comprehensive reporting of ethical safeguards enables readers to evaluate whether human-AI collaboration was conducted responsibly, transparently, and in accordance with accepted scientific and ethical standards.

#### Limitations of AI use

Finally, investigators should acknowledge limitations associated with AI-assisted inquiry, including model limitations, computational uncertainty, epistemological constraints, prompt dependence, and circumstances in which human interpretation remained essential. transparent discussion of these limitations strengthens rather than weakens methodological credibility by clarifying the scope and boundaries of AI participation. Therefore, comprehensive reporting of AI limitations enables readers to evaluate the appropriateness of AI participation, the robustness of the resulting meta-inferences, and the boundaries within which the study’s conclusions should be interpreted.

### Reporting standards for AI-augmented transformatizing

Table 3 presents the Reporting Standards for AI-Augmented Transformatizing in Human-AI Augmented Mixed Methods Research. The framework organizes reporting requirements into six domains corresponding to the major components of recursive Human-AI inquiry. Within each domain, specific reporting items identify the information necessary to support methodological transparency, recursive traceability, accountability, reproducibility, and critical appraisal.

**TABLE 3:** Reporting standards for Artificial intelligence (AI)-augmented transformatizing in human-AI augmented mixed methods research.

Domain	Reporting standard	What should be reported	Rationale
AI system disclosure	AI platform	Name of AI platform or service	Identifies computational environment
	Developer	Organization responsible for AI system	Enables methodological transparency
	Model family	GPT, Claude, Gemini, etc.	Documents analytic capabilities
	Model version	Exact version or release	Supports interpretability
	Access dates	Dates AI was used	Accounts for model evolution
	Configuration	API/web interface, memory status, custom instructions, temperature (where applicable), multimodal features	Documents computational context
Prompt documentation	Analytic role	Specific analytic functions performed by AI	Clarifies Human–AI division of labor
	Prompt development	How prompts were designed	Demonstrates methodological alignment
	Prompt evolution	How prompts changed during recursive inquiry	Documents recursive learning
	Prompt traceability	Complete prompt history	Supports transparency
	Recursive prompting	Number and purpose of recursive prompt cycles	Demonstrates iterative analysis
	Context supplied	Data, theory, coding framework, memos supplied to AI	Clarifies representational context
Human–AI interaction	Interaction cycles	Sequence of Human–AI analytic exchanges	Documents recursive collaboration
	Researcher interventions	Human critiques, revisions, contextualization	Demonstrates human oversight
	Acceptance/rejection/modification	Decisions regarding AI-generated representations	Supports representational adjudication
	Recursive decisions	Major methodological decisions across cycles	Demonstrates recursive reasoning
	Human interpretive authority	Points at which humans retained final judgment	Reinforces scholarly responsibility
Validation and auditability	Documentation	Records of analytic procedures	Facilitates methodological evaluation
	Version history	Major revisions across recursive inquiry	Documents methodological evolution
	Recursive audit trail	Complete chronological analytic record	Enables auditability
	Validation procedures	Verification strategies used	Supports methodological rigor
	Reproducibility	Documentation supporting methodological reconstruction	Enhances transparency
Ethical reporting	Confidentiality	Data protection procedures	Protects participants
	Bias mitigation	Identification and management of AI bias	Enhances fairness
	Hallucination verification	Procedures for detecting fabricated AI outputs	Protects scientific integrity
	Responsible AI use	Appropriate methodological use of AI	Supports ethical scholarship
	Human accountability	Statement of human responsibility for findings	Reinforces interpretive authority
Limitations	Model limitations	Known AI constraints	Supports balanced interpretation
	Uncertainty	Remaining ambiguities	Encourages cautious inference
	Epistemological limits	Boundaries of AI-generated knowledge	Reinforces human epistemology
	Appropriate caveats	Limits of transferability and interpretation	Prevents overgeneralization

The reporting standards are intended to *complement* existing methodological guidelines rather than to replace them. For example, qualitative research studies should continue to follow standards such as COREQ or SRQR where appropriate, systematic reviews should continue to report according to standards such as PRISMA, and experimental investigations should continue to follow standards such as CONSORT. Table 3 extends these established frameworks by documenting methodological elements unique to recursive Human-AI collaboration.

### Using the reporting standards

The reporting standards should be interpreted as an integrated framework rather than as a checklist to be completed only during manuscript preparation. Ideally, investigators should use the framework prospectively throughout the research process to document AI systems, prompts, recursive interactions, methodological decisions, validation activities, and ethical safeguards as they occur. Such documentation strengthens methodological transparency while reducing the likelihood that important details will be omitted during manuscript preparation.

Likewise, reviewers and editors should interpret the reporting standards holistically. The reporting domains are mutually reinforcing rather than independent. Comprehensive AI system disclosure has limited value without documentation linking prompts to AI-generated outputs; iterative interaction cannot be evaluated without documentation of researcher interventions; and methodological accountability depends on transparent reporting of validation procedures, ethical safeguards, and AI limitations. Consequently, transparent reporting emerges through the combined application of all six reporting domains rather than through isolated compliance with individual reporting items.

The reporting standards proposed in this article complement the broader methodological architecture developed throughout the manuscript. Building on the AI-Augmented Transformatizing Protocol for conducting recursive Human-AI analysis and the Human-AI Augmented Mixed Methods Quality Assessment Matrix (HAAM-QAM) for evaluating methodological rigor, the present section introduces reporting standards designed to promote transparency, reproducibility, accountability, and methodological scrutiny in Human-AI Augmented Mixed Methods Research. Together, the AI-Augmented Transformatizing Protocol, the HAAM-QAM, and the reporting standards constitute an integrated methodological framework for conducting, evaluating, and reporting Human-AI Augmented Mixed Methods Research.

The integrated methodological framework is illustrated through a worked example demonstrating how recursive Human-AI collaboration supports the progression from representational preparation to meta-inference construction.

## Worked example: applying the AI-augmented transformatizing protocol

### Purpose and overview of the worked example

The preceding sections established an integrated methodological architecture for Human-AI Augmented Mixed Methods Research by presenting the AI-Augmented Transformatizing Protocol, the Human-AI Augmented Mixed Methods Quality Assessment Matrix (HAAM-QAM), and reporting standards for transparent Human-AI collaboration. The methodology is now illustrated through a worked example demonstrating how these complementary components operate together during a single analytic investigation.

The worked example is intended as a methodological demonstration rather than as an empirical research study. All materials used in this worked example, including the four interview excerpts attributed to graduate students, are hypothetical and were constructed by the author solely for methodological illustration; they were not collected from actual human participants or derived from an empirical study. Accordingly, the worked example involved no human-subjects research, participant recruitment, or collection of identifiable or private information and, therefore, did not require institutional ethics approval or informed consent. Its objective is to make the recursive logic of AI-Augmented Transformatizing visible by illustrating how human researchers and AI collaboratively generate, evaluate, refine, and integrate representations while preserving human interpretive authority throughout the analytic process. In so doing, the example translates the conceptual and methodological foundations developed throughout this article into a transparent illustration of Human-AI Augmented Mixed Methods Research in practice.

For illustrative purposes, the example begins with a small qualitative dataset consisting of interview excerpts from graduate students discussing their experiences using generative AI during mixed methods research and subsequently introduces a brief hypothetical quantitative representation to demonstrate mixed methods integration. The dataset was selected because it contains both convergent and divergent perspectives regarding AI-assisted inquiry while remaining sufficiently concise to demonstrate each phase of the protocol. The substantive findings themselves are not the focus of the example; rather, the emphasis is on demonstrating the recursive methodological procedures through which Human-AI collaboration contributes to transparent and defensible meta-inference construction.

### Illustrative dataset

The illustrative dataset comprises four hypothetical, author-constructed interview excerpts representing possible reflections of graduate students using generative AI during research design, qualitative analysis, and scholarly writing. These hypothetical excerpts were designed to represent both opportunities and challenges associated with AI-assisted inquiry, including verification of AI-generated interpretations, prompt construction, recursive dialogue, and the continuing importance of human judgment. These excerpts were selected because they provide a sufficiently rich context for demonstrating recursive Human-AI collaboration while remaining concise enough to illustrate each stage of the AI-Augmented Transformatizing Protocol.

These excerpts served as the initial illustrative representations entering the AI-Augmented Transformatizing Protocol. The researcher first reviewed the data independently before initiating AI-assisted analysis, thereby establishing a transparent human interpretive baseline against which subsequent AI-generated representations could be evaluated. This initial human analysis ensured that AI contributions complemented rather than replaced researcher judgment, thereby preserving human interpretive authority throughout the recursive analytic process.

### Application of the AI-augmented transformatizing protocol

#### Phase 1: representational preparation

The hypothetical interview excerpts were organized into an illustrative analytic corpus, and contextual information needed for the methodological demonstration was documented. A preliminary analytic memorandum recorded the research purpose and theoretical orientation. These activities established the representational foundation for subsequent Human-AI collaboration.

**Output:** Human-reviewed qualitative corpus.

#### Phase 2: human analytic orientation

The researcher conducted an initial qualitative analysis through repeated reading, open coding, and preliminary thematic development. Reflexive memo writing documented the researcher’s initial assumptions and interpretive orientation before AI participation. This initial analytic orientation established a transparent human interpretive baseline against which subsequent AI-generated representations could be compared, evaluated, and refined.

Table 4 summarizes the initial human coding and the preliminary themes derived from the illustrative interview excerpts.

**TABLE 4:** Initial human codes and preliminary themes derived from the illustrative interview excerpts.

Illustrative excerpt	Initial human codes	Preliminary theme
Participant 1: AI helped identify patterns but prompted concern regarding prompt influence.	Pattern recognition; prompt influence; questioning AI interpretations	Prompt design influences representational development
Participant 2: AI generated useful ideas that required verification against original transcripts.	Verification; critical evaluation; comparison with data	Verification and critique strengthen AI-assisted analysis
Participant 3: disagreement with AI promoted deeper thinking.	Reflection; disagreement; recursive learning	Recursive dialogue enhances interpretive depth
Participant 4: AI should challenge assumptions rather than replace researcher judgment.	Human judgment; methodological responsibility; alternative perspectives	AI as an analytic catalyst rather than an analytic replacement

Initial human coding was conducted independently before AI-assisted analysis to establish the interpretive baseline for subsequent recursive Human–AI collaboration. All interview excerpts presented in this table are hypothetical, author-constructed examples created solely for methodological illustration; they were not collected from or attributed to actual human participants.

The preliminary analysis led to the identification of the following five tentative themes: (a) AI as an analytic catalyst rather than an analytic replacement; (b) human judgment as the foundation of trustworthy interpretation; (c) recursive dialogue enhances interpretive depth; (d) verification and critique strengthen AI-assisted analysis; and (e) prompt design influences representational development. These themes were intentionally treated as provisional representations that served as the starting point for subsequent recursive Human-AI refinement and meta-inference construction.

**Output:** Initial human codes and preliminary thematic representations.

#### Phase 3: AI-assisted transformation

The researcher next engaged an AI system using a documented prompt requesting identification of recurring concepts, preliminary themes, and conceptual relationships. The prompt specified that the AI should treat its response as a tentative analytic representation rather than as definitive findings. This approach positioned AI as an analytic participant contributing alternative representations for subsequent human evaluation, critique, and recursive refinement rather than as an autonomous source of interpretation.

The AI generated a thematic representation emphasizing AI as a cognitive partner, dialogic knowledge construction, critical verification, and continuing human agency. Although these themes overlapped substantially with the initial human interpretation, they also introduced alternative conceptual emphases that had not emerged during the first cycle of human analysis. These alternative representations served as the basis for subsequent recursive comparison, critique, and refinement, thereby expanding the range of interpretations available for meta-inference construction.

**Output:** AI-generated thematic representations.

#### Phase 4: human critique and contextualization

Consistent with the protocol, the researcher critically evaluated the AI-generated representation rather than accepting it uncritically. Evaluation focused on representational fidelity, contextual adequacy, conceptual completeness, and methodological transparency. This critical appraisal ensured that AI-generated representations informed, but did not determine, subsequent analytic decisions, thereby preserving human interpretive authority throughout the recursive inquiry.

Table 5 compares the initial human interpretation with the AI-generated representation.

**TABLE 5:** Comparison of human and Artificial intelligence (AI) representations following the first recursive cycle.

Analytic dimension	Human interpretation	AI-generated representation	Researcher evaluation
Primary analytic emphasis	Verification and researcher judgment	AI as a cognitive partner promoting reflective thinking	Complementary perspectives; both retained
Role of AI	Analytic assistant supporting interpretation	Dialogic collaborator stimulating metacognitive reflection	AI framing expanded conceptual understanding
Role of human researcher	Maintains interpretive authority and validates findings	Governs interpretation while engaging AI collaboratively	Strong convergence
Prompt construction	Recognized as influencing analysis	Mentioned only implicitly	Identified as underdeveloped in AI representation
Recursive dialogue	Present but secondary	Central organizing concept	Expanded in subsequent recursive cycle
Overall assessment	Strong methodological foundation	Greater conceptual abstraction but required contextual refinement	Human critique informed revised prompting

Human evaluation focused on representational fidelity, contextual adequacy, conceptual completeness, and methodological transparency using the Human-AI Augmented Mixed Methods Quality Assessment Matrix (HAAM-QAM).

The comparison demonstrated substantial convergence regarding the continuing importance of human interpretive authority while also revealing complementary conceptual emphases. Specifically, the AI-generated representation emphasized dialogic learning and cognitive partnership, whereas the initial human interpretation placed greater emphasis on prompt construction, verification, and methodological responsibility. Rather than viewing these differences as analytic inconsistencies, the researcher interpreted them as complementary perspectives that informed subsequent recursive refinement and meta-inference construction.

**Output:** Critically evaluated and contextually validated representations.

#### Phase 5: recursive re-prompting and re-representation

Based on the human critique, the researcher revised the prompt to focus explicitly on prompt construction, representational disagreement, and recursive methodological learning. The revised AI response incorporated these concerns by emphasizing recursive learning, constructive representational tension, and human interpretive governance. This recursive exchange demonstrated how successive cycles of Human-AI interaction can refine analytic representations by integrating complementary perspectives while preserving human interpretive authority.

This second cycle illustrates one of the defining characteristics of AI-Augmented Transformatizing: later prompts emerge directly from evaluation of earlier representations, allowing analytic understanding to evolve through successive cycles of Human-AI interaction rather than through repeated independent analyses. Consequently, recursive prompting functions as a methodological mechanism for progressively refining representations and strengthening the evidentiary basis for meta-inference construction.

**Output:** Recursively refined Human-AI representations.

#### Phase 6: meta-inference construction

The researcher integrated the initial human interpretation, the first AI-generated representation, the documented human critique, and the refined AI representation into a single explanatory framework. Rather than privileging any single representation, the researcher synthesized areas of convergence and divergence to construct a comprehensive meta-inference that reflected the complementary contributions of both human and AI analysis. This final stage demonstrates that meta-inferences emerge through recursive representational integration guided by human interpretive authority rather than through either independent human analysis or autonomous AI-generated interpretation.

Table 6 summarizes the evolution of analytic representations across successive cycles of recursive Human-AI collaboration.

**TABLE 6:** Evolution of representations across recursive human–Artificial intelligence (AI) analytic cycles.

Analytic stage	Primary representation	Major contribution	Influence on subsequent cycle
Phase 2: human analytic orientation	Initial human coding and preliminary themes	Established interpretive baseline and reflexive orientation	Provided foundation for AI prompting
Phase 3: AI-assisted transformation	AI representation 1	Introduced alternative thematic organization emphasizing cognitive partnership and dialogic learning	Prompted systematic human critique
Phase 4: human critique	Researcher evaluation of AI Representation 1	Identified strengths, contextual limitations, and omitted concepts	Guided revision of prompts
Phase 5: recursive re-prompting	AI representation 2	Emphasized recursive methodological learning, prompt construction, and representational tension	Produced refined conceptual framework
Phase 6: meta-inference construction	Integrated human–AI representation	Synthesized complementary strengths into a unified explanatory framework	Generated final meta-inference
Phase 7: reflexive audit	Recursive audit trail	Documented prompt evolution, researcher decisions, and representational provenance	Supported methodological transparency and reproducibility

The progression illustrates the recursive nature of AI-Augmented Transformatizing, in which each analytic cycle builds upon the evaluation of preceding representations rather than replacing them.

The resulting meta-inference was that the methodological value of AI does not arise from replacing human interpretation but from expanding the representational space available for inquiry through recursive cycles of prompting, critique, refinement, and integration. Human researchers remained responsible for evaluating competing representations, resolving conceptual tensions, and constructing the final explanatory interpretation. Accordingly, this worked example illustrates that robust meta-inferences emerge through recursive integration of complementary human- and AI-generated representations rather than through either independent human analysis or autonomous AI-generated interpretation.

**Output:** Integrated Human-AI meta-inference.

##### Brief mixed methods extension

In order to demonstrate how the same recursive process extends beyond qualitative material, the worked example can be expanded hypothetically to include a quantitative representation. Suppose that the four illustrative interview excerpts formed the qualitative component of a larger mixed methods research study in which graduate students also completed a 10-point item assessing perceived usefulness of generative AI for research. For methodological illustration, assume that the quantitative results indicated generally favorable perceptions of AI usefulness (*M* = 7.4, SD = 1.6), but with some variability across respondents. These hypothetical statistics are author-constructed solely for illustrating mixed methods integration and do not represent empirical observations.

During Phase 6, the quantitative representation would be compared with the recursively refined qualitative representations through a joint display. The relatively high mean would converge with qualitative themes portraying AI as an analytic catalyst and cognitive partner, whereas the variability in ratings would complement qualitative accounts emphasizing that AI usefulness depends on prompt quality, verification, contextual judgment, and continued human oversight. Rather than interpreting the quantitative finding independently, the researcher could prompt the AI system to identify possible relationships between the statistical pattern and the qualitative themes, to evaluate critically the resulting AI-generated integration, and to revise the integrated interpretation where necessary. This process would yield the following meta-inference: graduate students can perceive generative AI as useful for research while simultaneously regarding its methodological value as conditional on iterative prompting, critical verification, and human interpretive governance. Thus, the quantitative and qualitative representations contribute different but complementary evidence to an integrated conclusion that neither representation supports as fully in isolation.

Table 7 illustrates this integration by aligning the hypothetical quantitative representation with the corresponding qualitative representations, identifying whether the relationship between them reflects convergence, complementarity, or expansion, and presenting the integrated interpretation generated from each comparison. In this way, the joint display makes visible how distinct quantitative and qualitative representations can be brought together systematically during meta-inference construction rather than being interpreted as parallel but disconnected findings.

**TABLE 7:** Illustrative integration of hypothetical qualitative and quantitative representations.

Quantitative representation	Qualitative representation	Relationship	Integrated interpretation
Perceived usefulness of generative AI was generally favorable (*M* = 7.4, SD = 1.6).	AI was represented as an analytic catalyst and cognitive partner.	Convergence	Both representations suggest that AI can contribute positively to research activity.
Ratings showed meaningful variability.	AI usefulness was described as dependent on prompt construction, verification, contextual judgment, and human oversight.	Complementarity	Perceived usefulness is conditional rather than uniform and depends on how Human-AI collaboration is structured and governed.
Favorable average rating accompanied by individual variation.	Recursive dialogue and critical verification were portrayed as strengthening AI-assisted analysis.	Expansion	The integrated evidence suggests that AI’s methodological value lies not merely in its availability, but in critically supervised and iterative use.

As shown in Table 7, the favorable mean rating converges with the qualitative characterization of AI as an analytic catalyst and cognitive partner, whereas variability in the quantitative ratings complements the qualitative finding that AI usefulness depends on prompt construction, verification, contextual judgment, and human oversight. The joint display further expands these findings by showing that AI’s methodological value is associated not merely with perceived usefulness but with the conditions under which Human-AI collaboration is structured and governed. Thus, Table 7 illustrates how quantitative results, qualitative themes, and AI-assisted integrative interpretation can enter the same recursive analytic process while human researchers retain responsibility for evaluating relationships among representations and constructing the final meta-inference.

#### Phase 7: reflexive audit and validation

The final phase documented the complete recursive analytic process. Prompt histories, AI-generated representations, reflexive memoranda, researcher decisions, recursive revisions, and representational evolution were preserved within an audit trail. This documentation demonstrated that prompts and analytic iterations were traceable and transparent, while enabling readers, reviewers, and editors to evaluate the integrity and trustworthiness of the complete Human-AI analytic process. In so doing, the audit trail completed the AI-Augmented Transformatizing Protocol by ensuring that every stage of recursive Human-AI collaboration could be reconstructed, critically appraised, and reported transparently.

**Output:** Recursive methodological audit trail.

### Lessons from the worked example

The worked example demonstrates several important characteristics of AI-Augmented Transformatizing. More importantly, it illustrates how recursive Human-AI collaboration transforms the protocol, quality framework, and reporting standards into an integrated methodological system for mixed methods research.

First, AI generated alternative representations rather than authoritative conclusions. Throughout the analysis, AI functioned as an analytic participant whose representations expanded the range of possible interpretations without replacing human judgment.

Second, human critique strengthened rather than constrained the analytic process. Systematic evaluation of AI-generated representations identified conceptual strengths, methodological limitations, and opportunities for recursive refinement that contributed directly to the development of more comprehensive meta-inferences.

Third, recursive prompting was shown to be substantially more informative than one-time prompting. Each successive interaction built explicitly on the evaluation of preceding representations, illustrating that Human-AI collaboration functions most effectively as an iterative process rather than as a single computational event.

Finally, the worked example demonstrates both recursive Human-AI analysis within a qualitative research strand and, through the abbreviated mixed methods research extension, the integration of qualitative and quantitative representations into a higher-order meta-inference. In particular, the joint display illustrates how convergence and complementarity across data types can be examined explicitly while AI-generated integrative interpretations remain subject to human critique and methodological governance. The protocol structured recursive inquiry, the HAAM-QAM guided methodological evaluation, and the reporting framework ensured transparent documentation of every stage of Human-AI collaboration.

The purpose of this worked example has not been to validate the AI-Augmented Transformatizing Protocol empirically but to demonstrate its practical implementation. Accordingly, the four hypothetical excerpts, two recursive cycles, and abbreviated quantitative extension are sufficient to make the protocol’s recursive and integrative logic visible, but they cannot represent the greater complexity likely to arise in empirical applications involving larger and more heterogeneous datasets, multiple qualitative and quantitative representations, extended Human-AI interaction histories, or additional cycles of critique and refinement. Together with the preceding sections, the example illustrates that Human-AI collaboration can be organized through transparent, recursive, and methodologically accountable procedures that preserve human interpretive authority while leveraging AI to generate richer representational possibilities for mixed methods inquiry.

## Implications for psychological science

### Methodological implications for psychological science

The integrated methodological framework developed throughout this article has implications that extend beyond Human-AI Augmented Mixed Methods Research itself. As AI becomes increasingly integrated into literature synthesis, qualitative analysis, quantitative interpretation, mixed methods integration, and scholarly communication, psychological science faces a broader methodological challenge: how to govern Human-AI collaboration in ways that preserve scientific rigor, transparency, accountability, and ethical responsibility (Bender et al., 2021; Grimmer et al., 2022).

The AI-Augmented Transformatizing Protocol, the Human-AI Augmented Mixed Methods Quality Assessment Matrix (HAAM-QAM), and the reporting standards proposed in this article address this challenge by providing an integrated methodological architecture for conducting, evaluating, and documenting recursive Human-AI inquiry. Rather than conceptualizing AI as either an autonomous analytic agent or merely a computational tool, this framework positions AI as an analytic participant whose contributions require continuous human evaluation and methodological oversight. Consequently, attention shifts from technological capability to methodological governance, emphasizing that the scientific value of AI depends on the transparency, rigor, and accountability of the processes through which AI-generated representations are critically evaluated and incorporated into empirical inquiry.

More broadly, the framework suggests that methodological innovation should be guided by enduring scientific principles rather than by the changing capabilities of individual AI systems. As computational technologies continue to evolve, psychological science will require methodological frameworks that remain applicable across successive generations of AI while preserving the discipline’s enduring commitments to transparency, reproducibility, reflexivity, and responsible scholarship.

### Implications for research practice

The framework proposed in this article has important implications for researchers, reviewers, journal editors, and scholarly publishers. For researchers, the principal implication is that AI-assisted inquiry should be approached as a transparent, recursively documented methodological process rather than as a series of isolated computational tasks. Researchers should document AI systems, prompting procedures, iterative interactions, methodological decisions, and human evaluations throughout the research process so that readers can understand how AI contributed to the development, evaluation, and integration of evidence culminating in defensible integrated conclusions.

For reviewers and editors, methodological evaluation should focus less on whether AI was used and more on how AI participated in the research process. Transparent documentation of prompts, iterative interactions, analytic development, and human oversight provides a stronger basis for evaluating methodological rigor than simply disclosing that AI assisted with analysis. The HAAM-QAM and the reporting standards proposed in this article provide complementary frameworks for assessing whether AI participation was transparent, methodologically justified, and consistently governed by human interpretive authority (Hallinger, 2014; Hong and Pluye, 2019).

These developments also have important implications for editorial policy. Journal editors increasingly may require explicit disclosure of AI systems, documentation of prompting procedures, preservation of methodological audit trails, and reporting of recursive Human-AI collaboration. Such expectations would extend existing commitments to transparency and reproducibility while promoting greater consistency in the evaluation of AI-assisted research. Importantly, these requirements should complement established reporting guidelines such as PRISMA, CONSORT, COREQ, and SRQR rather than replace them, thereby extending existing standards of scientific reporting to accommodate transparent Human-AI collaboration (Page et al., 2021; Tong et al., 2007).

### Implications for graduate education and ethical AI use

Graduate education represents another important context in which the integrated methodological framework proposed in this article may have lasting influence. As AI becomes increasingly available to students and early-career researchers, graduate programs should move beyond teaching the mechanics of AI use toward developing competencies in recursive Human-AI collaboration. Specifically, students should learn to formulate theoretically informed prompts, to evaluate critically AI-generated representations, to document iterative analytic processes, to recognize algorithmic limitations, and to maintain responsibility for methodological interpretation throughout the research process (Leonelli, 2019; Mittelstadt et al., 2016). Preparing researchers in these competencies will be essential for ensuring that future AI-assisted psychological research remains transparent, methodologically rigorous, and ethically accountable.

Equally important, graduate education should continue to emphasize methodological reflexivity. Human-AI Augmented Mixed Methods Research extends traditional reflexive practice by encouraging researchers to examine not only how their own assumptions influence inquiry, but also how prompt design, alternative AI-generated representations, and recursive Human-AI interactions shape emerging interpretations. Such reflexivity strengthens rather than diminishes critical thinking because it promotes continual evaluation of both human and computational contributions to knowledge construction.

Ethical AI use likewise requires preserving human interpretive authority throughout recursive inquiry. Although AI systems can generate conceptually rich representations and identify alternative analytic perspectives, responsibility for methodological decisions, interpretation, and published conclusions remains with human researchers. Therefore, ethical AI-assisted inquiry requires transparency regarding AI participation, critical evaluation of algorithmic bias, protection of participant confidentiality, and explicit acknowledgment of the limitations of AI-generated representations (Barocas et al., 2023; Bender et al., 2021). These principles reinforce the central argument advanced throughout this article: responsible Human-AI collaboration depends on methodological governance rather than on technological capability.

### Looking forward

The integration of AI into psychological science is likely to continue accelerating as computational systems become increasingly sophisticated and accessible. Future developments may expand AI’s capacity to contribute to theory generation, evidence synthesis, multimodal analysis, and interdisciplinary collaboration. Nevertheless, the methodological questions addressed throughout this article are unlikely to diminish in importance. Researchers will continue to require guidance on how AI-generated representations should be evaluated, how recursive Human-AI interactions should be documented, and how methodological accountability should be maintained as AI assumes an increasingly prominent role in scientific inquiry. Accordingly, the need for principled methodological frameworks that govern Human-AI collaboration is likely to increase rather than diminish as AI capabilities continue to evolve.

The integrated methodological framework proposed in this article suggests that future methodological development should focus not only on improving AI technologies, but also on strengthening the principles that govern their responsible use. Transparency, reflexivity, accountability, reproducibility, and human interpretive authority are enduring scientific values that should remain central irrespective of advances in computational capability. By grounding Human-AI collaboration in these principles, psychological science can encourage methodological innovation while preserving the rigor, transparency, and trustworthiness upon which empirical inquiry depends.

Having established the methodological implications of Human-AI Augmented Mixed Methods Research, the discussion synthesizes the principal contributions of the AI-Augmented Transformatizing framework, considers its limitations, and identifies priorities for future methodological development and empirical evaluation.

## Discussion

This article translates AI-Augmented Transformatizing into a practice-oriented methodological framework for conducting, evaluating, and reporting Human-AI Augmented Mixed Methods Research. Its central contribution is to shift attention from whether AI participates in research to how that participation can be structured, scrutinized, documented, and governed while human researchers retain interpretive and ethical responsibility. Therefore, the following discussion focuses on the framework’s distinctive methodological contributions, limitations, and priorities for empirical evaluation rather than restating its preceding theoretical and procedural components.

### Principal contributions

The integrated methodological framework presented in this article makes three principal contributions to methodological scholarship. First, it operationalizes AI-Augmented Transformatizing by translating a conceptual framework into an operational methodology. Whereas the companion article established the philosophical foundations of representational-recursive inquiry, the present article specifies how recursive Human-AI collaboration can be implemented through the AI-Augmented Transformatizing Protocol. By providing explicit procedures for representational preparation, human analytic orientation, AI-assisted transformation, human critique and contextualization, recursive re-prompting and re-representation, meta-inference construction, and reflexive audit and validation, the protocol offers researchers a transparent and systematic methodology for incorporating AI into mixed methods research while preserving human interpretive authority, methodological transparency, and scientific accountability.

Second, the article introduces an integrated framework for methodological governance. Rather than viewing AI as either an autonomous analyst or merely a computational tool, the framework conceptualizes AI as an analytic participant whose contributions require continuous human evaluation, documentation, and critical interpretation. This perspective is operationalized through the HAAM-QAM, which evaluates the integrity of representations and Human-AI analysis, the transparency of iterative analytic development, the preservation of human oversight, and the quality and ethical accountability of the resulting integrated conclusions. Complementing the HAAM-QAM, the reporting standards proposed in this article establish practical expectations for documenting recursive Human-AI collaboration in a transparent, reproducible, and methodologically accountable manner.

Third, the article contributes to the continuing evolution of mixed methods methodology by extending the concept of integration beyond the combination of qualitative and quantitative evidence. Human-AI Augmented Mixed Methods Research conceptualizes integration as a recursive process in which human-generated and AI-generated representations are compared, critiqued, refined, and synthesized through successive cycles of analysis. In so doing, the framework expands the methodological scope of mixed methods research by incorporating recursive Human-AI collaboration as an additional source of integration while remaining consistent with the field’s longstanding emphasis on integration, complementarity, and the construction of defensible meta-inferences (Corrigan and Onwuegbuzie, 2020; Polyviou et al., 2024; Schoonenboom, 2022, 2023; Shannon-Baker and Hunt-Anderson, 2025; Tashakkori and Teddlie, 1998; Younas and Durante, 2022, 2023; Younas et al., 2023, 2025).

Collectively, these contributions shift the focus of AI-assisted research from computational capability to methodological governance. The long-term value of AI within psychological science will depend less on the sophistication of individual models than on the transparency, accountability, and rigor with which Human-AI collaboration is conducted and evaluated. Accordingly, methodological frameworks that govern responsible Human-AI collaboration will become increasingly essential as AI continues to evolve and to assume a more prominent role in psychological research.

### Limitations

Despite its contributions, the integrated methodological framework proposed in this article has several important limitations that should inform its interpretation and guide future refinement. The first set of limitations is methodological. Human-AI Augmented Mixed Methods Research continues to depend fundamentally on researcher expertise. AI systems may generate alternative representations, but researchers remain responsible for formulating prompts, for evaluating representational fidelity, for resolving competing interpretations, and for constructing defensible meta-inferences. Consequently, the quality of AI-assisted inquiry remains closely linked to the methodological competence, theoretical sensitivity, and disciplinary expertise of the researcher (Lincoln and Guba, 1985; Saldaña, 2025). Moreover, the worked example presented in this article is intended to illustrate the operational implementation of the framework rather than to provide empirical evidence of its effectiveness. Although it demonstrates the operational logic of the framework, empirical studies are needed to determine its effectiveness across different research designs, substantive disciplines, and methodological traditions.

A second group of limitations arises from the evolving nature of contemporary AI systems. LLMs continue to change through updates to model architecture, training data, and deployment procedures, meaning that identical prompts may produce different outputs over time. Furthermore, AI-generated representations remain susceptible to hallucinations, incomplete contextual understanding, and algorithmic bias (Bender et al., 2021; Mittelstadt et al., 2016). Accordingly, AI-generated representations always should be regarded as provisional and subject to systematic human evaluation. Although the framework proposed in this article is designed to mitigate these challenges through recursive critique, prompt traceability, and methodological documentation, no methodological framework can eliminate them entirely.

Finally, important ethical considerations remain. Responsible Human-AI collaboration requires careful attention to participant confidentiality, transparency regarding AI participation, appropriate acknowledgment of AI limitations, and continued human responsibility for methodological decisions and published conclusions. Therefore, ethical governance should be understood as an ongoing process of professional judgment rather than as a technical property of AI systems themselves. Although the framework presented in this article supports such judgment through explicit procedures for documentation, evaluation, and reporting, it cannot replace the ethical responsibilities of researchers.

These limitations should not be interpreted as weaknesses unique to AI-Augmented Transformatizing. Rather, they reflect the realities of conducting research within a rapidly evolving technological environment and reinforce the need for explicit methodological guidance governing Human-AI collaboration. Accordingly, the framework should be viewed as an evolving methodological foundation that can be refined and extended as both AI technologies and methodological knowledge continue to develop.

### Future directions

The framework presented in this article establishes a foundation for a broader program of methodological research. Its long-term value ultimately will depend on continued empirical evaluation, theoretical refinement, and practical application across diverse research contexts. As AI technologies and research practices continue to evolve, the framework likewise should evolve through ongoing methodological scholarship and empirical investigation.

A first priority is systematic validation of the methodological framework itself. Future studies should examine whether the AI-Augmented Transformatizing Protocol improves analytic transparency, representational fidelity, methodological rigor, and the quality of meta-inferences when compared with conventional approaches to mixed methods analysis. Similarly, the Human-AI Augmented Mixed Methods Quality Assessment Matrix (HAAM-QAM) and the reporting standards proposed in this article require systematic empirical evaluation to determine their validity, reliability, usability, and value for researchers, reviewers, and editors. For the HAAM-QAM specifically, priorities include examining whether its proposed three-domain, eight-dimension structure demonstrates construct-related validity and whether independent researchers, reviewers, and editors can apply its dimensions and performance descriptors consistently, thereby establishing inter-rater reliability across different methodological and substantive contexts. Such evaluation should include comparisons across different research designs, methodological traditions, substantive disciplines, and AI platforms to determine the generalizability and practical utility of the proposed framework.

A second priority involves extending the framework across disciplinary and technological contexts. Although developed primarily for Human-AI Augmented Mixed Methods Research in psychology, its underlying methodological principles are potentially applicable across education, the health sciences, organizational research, sociology, communication studies, and other disciplines in which AI increasingly contributes to empirical inquiry. Future research also should examine how the AI-Augmented Transformatizing Protocol functions across different AI models, LLMs, and emerging multimodal systems while preserving the methodological principles of transparency, accountability, and human interpretive authority that define the framework.

Finally, future research should examine the educational and professional implications of Human-AI collaboration. Graduate education, reviewer training, and professional development increasingly will require evidence-based approaches for preparing researchers to collaborate responsibly with AI in scientific inquiry. Such efforts should emphasize not only technical proficiency, but also methodological reasoning, critical evaluation, ethical judgment, and recursive reflexivity. Ultimately, preparing researchers to collaborate effectively with AI may turn out to be as important as continuing to improve AI systems themselves.

Taken together, these future directions suggest that AI-Augmented Transformatizing should be viewed not as a completed methodology, but as the foundation of an evolving methodological research program. As AI technologies continue to advance, the framework presented in this article provides a principled basis for ensuring that Human-AI collaboration remains transparent, accountable, methodologically rigorous, and scientifically trustworthy. In this way, the framework is intended not only to guide current practice, but also to support the continued evolution of Human-AI Augmented Mixed Methods Research as a principled and enduring methodological approach.

## Concluding synthesis

As AI becomes increasingly involved in psychological and mixed methods research, the central methodological question is how Human-AI collaboration can be conducted transparently, rigorously, and responsibly. This article addresses that question through three integrated contributions: the AI-Augmented Transformatizing Protocol for structuring Human-AI analysis, the HAAM-QAM for evaluating methodological quality, and reporting standards for documenting AI participation. Together, these components provide a practical framework for conducting, evaluating, and reporting Human-AI Augmented Mixed Methods Research.

The central contribution is methodological rather than technological. AI-generated analyses are treated as provisional contributions to inquiry that require critical human evaluation, while researchers retain responsibility for methodological decisions, ethical judgment, interpretation, and final integrated conclusions. In this way, the framework seeks to make AI participation visible and critically appraisable without displacing established principles of rigorous mixed methods inquiry.

The framework should be regarded as an evolving methodological proposal rather than as a completed or empirically validated system. Future research should test, refine, and adapt its components across research designs, disciplines, datasets, and AI systems while evaluating the validity, reliability, usability, and practical consequences of its quality criteria. Such work will determine the extent to which the framework can support transparent and trustworthy Human-AI collaboration as research practices continue to evolve.

## Data Availability

The original contributions presented in this study are included in the article/supplementary material, further inquiries can be directed to the corresponding author.
